# Sarcoidosis in a dental surgeon: a case report

**DOI:** 10.1186/1752-1947-4-259

**Published:** 2010-08-10

**Authors:** Luigi Checchi, Maria C Nucci, Antonietta M Gatti, Daniele Mattia, Francesco S Violante

**Affiliations:** 1 Department of Periodontology and Implantology, School of Dentistry, University of Bologna, Italy; 2 Occupational Medicine Unit, S. Orsola-Malpighi Hospital, Bologna, Italy; 3 CNISM, Laboratory of Biomaterials, Department of Neurosciences, University of Modena and Reggio Emilia, Italy

## Abstract

**Introduction:**

Although the causes of sarcoidosis are still unknown, past and current studies have provided evidence that this disease may be associated with occupational exposure to specific environmental agents. We describe a case of sarcoidosis in a dental surgeon with long exposure to inorganic dusts. To the best of our knowledge, this is the first report of this kind in the literature.

**Case presentation:**

At the beginning of 2000, a 52-year-old Caucasian man, who worked as a dental surgeon, presented with shortness of breath during exercise, cough and retrosternal pain. After diagnosis of sarcoidosis, a scanning electronic microscopy with X-ray microanalysis of biopsy specimens was used in order to determine whether the disease could be traced to an occupational environmental agent. Results showed the presence of inorganic dust particles within sarcoidotic granulomas, and demonstrated that the material detected was identical to that found in a powder used by our patient for several years.

**Conclusions:**

Although these results cannot be considered as definitive proof, they do however provide strong evidence that this disease may be associated with material used by dental surgeons.

## Introduction

Past and current studies suggest that environmental and occupational factors may be associated with increased risk of sarcoidosis. Sarcoidosis is a systemic granulomatous disease that primarily affects the lung and lymphatic systems of the body [1-3]. Although its causes are still unknown, different lines of evidence associate sarcoidosis with exposure of genetically susceptible hosts to specific environmental agents. The possible agents involved in the etiology of the disease include micro-organisms, organic and inorganic dusts such as aluminum, zirconium, talc [1], titanium and silicates. We present a case of sarcoidosis in which inorganic dust particles (silicate) were detected in biopsy material by means of scanning electronic microscopy (ESEM) tandem energy dispersive spectrometer (EDS), thus providing additional evidence that some cases of sarcoidosis may be due to occupational exposure to environmental agents.

## Case presentation

At the beginning of 2000, a 52-year-old Caucasian man who worked as a dental surgeon and had never smoked, was referred to the our Occupational Health Unit, owing to shortness of breath during exercise, cough and retrosternal pain, which had become progressively evident in the months prior to admission. His medical history was unremarkable. Physical examination, pulmonary function tests and blood tests were within normal ranges. Chest X-ray showed bilateral hilar enlargement and suggested hilar lymphadenopathy secondary to sarcoidosis as a possible diagnosis. This possibility was supported by chest computed tomography (CT) scan, which revealed diffuse hilar and mediastinal lymphadenopathy with maximal lymph node diameter of 3 cm. Total body scintigraphy with gallium 67 confirmed the hilar and mediastinal lymphadenopathy. Bronchoalveolar lavage (BAL) showed an increase in total cell count and an elevated CD4/CD8 ratio compatible with sarcoidosis, and absence of neoplastic cells or infectious agents. Finally, cytologic examination of the aspirated bronchial material evidenced a non-caseating granuloma and first stage sarcoidosis was diagnosed. After lung examination, it was decided not to start any treatment and to evaluate the progression of the disease. Therefore, our patient was referred for follow-up.

Given the epidemiologic evidence on the role of occupational exposure in the etiology of sarcoidosis, a sample of the powder used by our patient in his work as a dental surgeon for at least 20 years in dental hygiene procedures was analyzed. Analysis was performed by electronic microscopy in the Laboratory of Biomaterial of the Department of Neurosciences, University of Modena and Reggio Emilia and identified the presence of silicates among components.

During the following three years, the clinical picture and chest CTs remained unchanged. Blood tests showed an increase of serum angiotensin converting enzyme (ACE) and GPT, whereas calcium remained within normal values. Hepatic echography was also normal. In 2003, our patient's dyspnea was seriously aggravated with episodes also occurring at rest; the chest CT performed in March 2003 revealed a bilateral increase in the number and volume of the affected mediastinal and hilar lymph nodes, whereas the remainder of the pulmonary parenchyma was normal. Consequently, treatment was initiated with cycles of cortisone, which produced visible improvement in the clinical picture, reduction of the lymphadenopathy on CT after seven months, and decline in serum ACE to within normal range. In 2006, while our patient's dyspnea remained under control with cortisone, CT showed progression of the disease with enlargement of the hilar and mediastinal lymphadenopathy and areas of interstitial thickening. Additional information about the possible origin of the disease was provided by SEM with X-ray microanalysis of biopsy specimens, which showed that the material found inside the sarcoidotic granulomas was identical to that found in the powder used by the dental surgeon for several years. Currently, our patient is working without using any dust for dental cleaning, his clinical picture is stable with daily cortisone treatment, and he is under periodic control.

## Discussion

The activity of dental surgeons is associated with airborne exposure to aerosols of biological material and inorganic substances. The instruments used during dental surgical procedures produce intense heat (electrocautery, laser) and such procedures usually produce fumes containing biological material (even partially incombusted). High-speed, air-driven dental hand-pieces, ultrasonic scalers, the polishing of composite and ceramic restorations and the use of the milling cutter on metallic prostheses disperse a large amount of aerosol and spatter including fine particulate matter [4]. During the polishing and whitening of natural teeth, chemical compounds (composed by particles of sodium di-carbonate and tricalcium phosphate) are sprayed on the tooth surface detaching part of its enamel and dispersing microparticles in the environment, thus exposing patients and operators at risk of inhalation. For example, a standard hygiene procedure involves the use of prophylactic material and ultrasonic and/or manual instrumentation [5]. The prophylaxis phase can be substituted by an artificial bicarbonate aerosol. Artificial cleaning aerosols are formed by a Mini-Clean device (Castellini, Bologna, Italy) with air pressure set at six to seven atmospheres and water flow at one atmosphere. Each individual run lasts 20 to 30 minutes for each patient. It has been reported that 95% of the particles measure less than five micrometers and are mainly concentrated within two meters of the patient where they can be easily inhaled by dental clinicians. For this reason, the patient always protected himself with a face mask (3 M ESPE 1942 F.B. (1800 NL) resistant molded face) whose level of efficiency was 90 to 92% [6,7].

**Figure 1:**
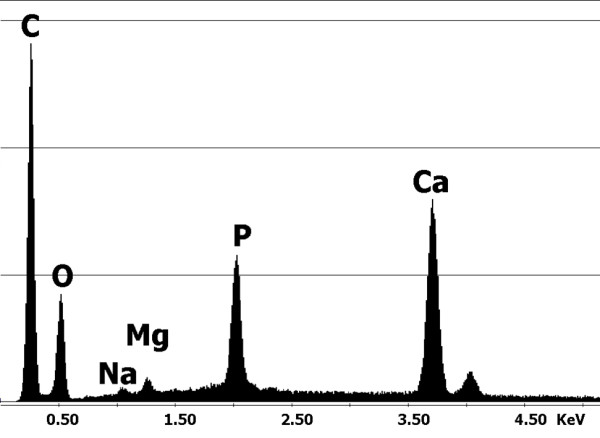
**Spectrum of the particles of calcium phosphate found in the lung biopsy**.

**Figure 2:**
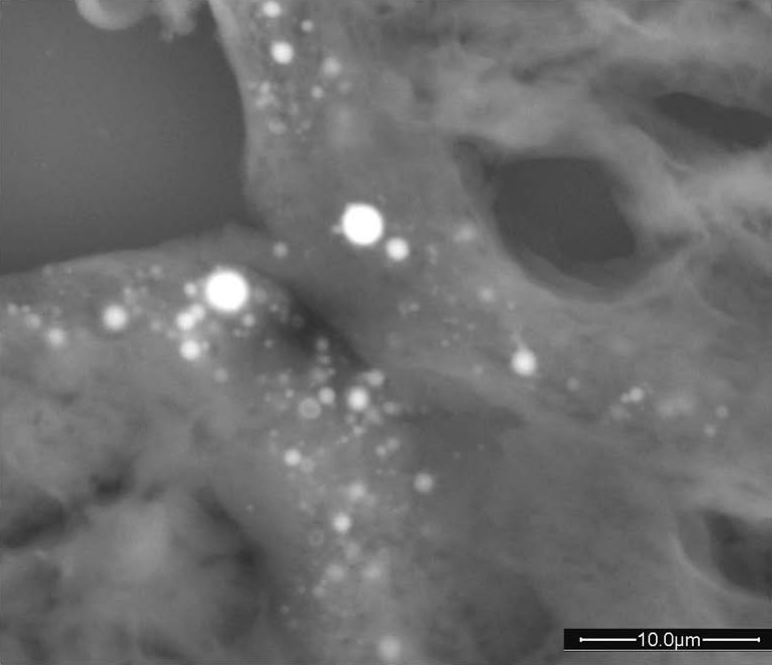
**Particles of calcium phosphate found in the lung biopsy**.

To test our hypothesis that sarcoidosis could be related to an environmental agent in this case, a further examination of biopsy specimens was performed. Sections of the lung biopsy were sent to the Laboratory of Biomaterials to be analyzed by Environmental Scanning Electron Microscopy. For elemental analyses, conducted by means of an Energy Dispersive Spectrometer (EDS by EDAX), the instrument was equipped with an X-ray microprobe. When debris was detected, a microanalysis was carried out to check their chemical composition. At the same time, and under the same working conditions, the inorganic chemical content of the biological tissues surrounding these particles was investigated and correlated to the chemistry of the particles. Considering our patient's long exposure to dust, a hypothesis was proposed that the particulate matter can originate from the wear of materials used during abrasion and polishing operations. It was put forward that the material used for dental cleaning procedures could have a similar chemistry to that of the found debris. A sample was observed under ESEM and analyzed by EDS. The spectrum obtained proved similar to that of the debris found in the lung (Figures 1 and 2).

## Conclusions

Although the relationship between dental material with silicates and sarcoidosis has not, to the best of our knowledge, been discussed in literature, these findings provide evidence that dental surgeons' occupational exposure to inorganic dusts might be a relevant factor in the onset of sarcoidosis. We think that similar cases should be reported in order to help identify environmental agents that could be involved in the pathogenesis of sarcoidosis.

## Consent

Written informed consent was obtained from the patient for publication of this case report and any accompanying images. A copy of the written consent is available for review by the Editor-in-Chief of this journal.

## Competing interests

The authors declare that they have no competing interests.

## Authors' contributions

MCN was the principal investigator, conceived the study and drafted and revised the manuscript. LC was responsible for considerations regarding dental materials and working procedures. AMG was responsible for electron microscopy analyses. LC was responsible for considerations regarding biomaterials and helped draft and revise the manuscript. FSV supervised the entire work and assumes responsibility for the entire manuscript. All authors read and approved the final manuscript.
